# Editorial: The Role of Optineurin in Immunity and Immune-Mediated Diseases

**DOI:** 10.3389/fimmu.2019.02803

**Published:** 2019-12-11

**Authors:** Andrew M. Smith, Folma Buss, Ivana Munitic

**Affiliations:** ^1^Microbial Diseases, Eastman Dental Institute, UCL, London, United Kingdom; ^2^Cambridge Institute for Medical Research, University of Cambridge, Cambridge, United Kingdom; ^3^Laboratory for Molecular Immunology, Department of Biotechnology, University of Rijeka, Rijeka, Croatia

**Keywords:** optineurin, innate immunity, inflammation, autophagy, vesicle trafficking

The multifunctional adaptor optineurin has been implicated in an increasing number of protein-protein interactions and cellular functions ever since its first identification as a binding partner for an adenoviral protein (1). Most—if not all—optineurin functions require its ubiquitin-binding domain in its C-terminus, which binds to K63- and/or M1-polyubiquinated proteins, allowing it to act, for example, as an adaptor during inflammatory signaling, autophagy, and vesicle trafficking (2–4). The interest in optineurin intensified after the identification of various mutations and polymorphisms in several human diseases, including primary open-angle glaucoma, amyotrophic lateral sclerosis (ALS), Paget's disease of the bone, and Crohn's disease. With their distinct yet unresolved pathogenesis, and complex genetic and environmental risk factors, these diseases seem unrelated at first. ALS, glaucoma, or Paget's disease are not traditionally regarded as immune-mediated diseases; however, the emerging evidence pinpoints immune system disfunction as their common denominator (5, 6). The aim of this Research Topic was to explore the role(s) of optineurin on a host of diverse cellular pathways that are directly or indirectly linked to the immune response. The articles cover immune signaling, cell death, membrane trafficking, autophagy of intracellular bacteria (xenophagy), damaged mitochondria (mitophagy), and protein aggregates.

In their mini review, Slowicka and van Loo give an overview of the various lines of evidence implicating optineurin in the regulation of the immune response. Due to its high homology to the NF-κB essential modulator (NEMO), optineurin was initially considered to be a negative regulator of NF-κB, a major proinflammatory transcription factor (7, 8). However, NF-κB hyperactivation was not identified in a range of primary cells from loss-of-function optineurin mouse models (9–12). This unexpected finding is further analyzed by Liu et al. through an investigation of the overexpression of the common optineurin ALS mutations in mouse embryonic fibroblasts; this is demonstrated to lead to higher NF-κB activation and inflammatory cytokine secretion. They follow this up by delivering lentiviruses carrying patient ubiquitin-binding deficient E478G mutation or wild-type optineurin into the mouse motor cortex, showing that expression of the mutant optineurin triggers immune cell infiltration, microgliosis, proinflammatory cytokine secretion, and impaired motor functions. Outlioua et al. review the role of optineurin in regulating antiviral type I interferon (IFN) production through activation of TANK-binding kinase 1 (TBK1) at the Golgi apparatus. TBK1 is K63-polyubiquitinated upon pathogen or damage-sensing, which allows TBK1 to be recognized and to dock onto optineurin by a bipartite interaction via its ubiquitin-binding site and coiled-coil regions. Optineurin thereby serves as an adaptor for TBK1 trans-autophosphorylation and activation. The authors also emphasize that the interaction between optineurin and TBK1 is multifaceted as optineurin itself becomes phosphorylated by TBK1, which is crucial for its role as an autophagy receptor and to its interaction with the microtubule-associated protein 1A/1B light chain 3B (LC3) in autophagosomal membranes.

Several publications elaborate on the role of optineurin in autophagy, which extends beyond its function as an autophagy receptor for the recruitment of LC3-positive membranes to ubiquitinated cargo. The central role of optineurin in diverse membrane trafficking pathways (autophagosome-lysosome fusion, vesicle secretion, etc.) and the importance of different binding partners is comprehensively summarized by Ryan and Tumbarello. This review specifically highlights the central role of optineurin in selective autophagy and how its disfunction is linked to neuronal cell death and neurodegeneration. Weil et al. have compiled an extensive review on mitophagy and the role of optineurin in mitochondrial regulation and its implications in neurodegeneration and cancer. Abnormalities in mitophagy are associated with a number of diseases, including Parkinson's disease, ALS, primary open angle glaucoma, and cancer. The current understanding on how optineurin mutations found in ALS and glaucoma impact on mitophagy are covered in the manuscript. Finally, the authors discuss whether the potential role of optineurin in mitophagy is also linked to other diseases, such as cancer and inflammatory bowel disease.

Toth and Atkin focus their review initially on the multiple cellular mechanisms of optineurin, and they then proceed to put these processes in context with ALS and glaucoma. Disease-associated optineurin mutations and the impact these have on the physiological processes of inflammatory signaling, endoplasmic reticulum stress, secretion, autophagy, and cell viability are discussed in detail. The review highlights the complexity of neurodegeneration and how optineurin in combination with other key disease-associated molecules, such as SOD1 and TBK1, is thought to contribute to disease progression. The review by Swarup and Sayyad specifically highlights the link between optineurin and glaucoma. The authors discuss the impact of the glaucoma-associated mutants, the E50K and M98K, on the cellular functions of optineurin. Interestingly, these glaucoma-specific mutations induce pathologies only in retinal cells despite optineurin being widely expressed in different cell types and tissues.

Tschurtschenthaler and Adolph's mini review brings together the current literature dealing with optineurin and intestinal inflammation and places them in the context of Crohn's disease etiology. Optineurin has been reported to play a major role in xenophagy, the regulation of endoplasmic reticulum stress, and the facilitation of pro-inflammatory cytokine secretion, all of which have a major impact on the intestine and have previously been associated with the development of Crohn's disease (13). The review covers findings from a number of studies that support an active role for optineurin in intestinal immunity and describe the chronic bowel inflammation that develops in its absence. Finally, they describe how optineurin limits the accumulation of an endoplasmic reticulum-anchored inflammatory signaling hub within the intestinal epithelium, which prevents the development of spontaneous enteritis. The precise role of optineurin in Crohn's disease is still unclear, and the authors thus highlight the need for further investigation.

This collection highlights the wide range of functions associated with optineurin, many of which are crucial for maintaining tissue homeostasis by use of the innate immune system. This may explain why optineurin mutations are found in a wide array of chronic disorders. Many open questions remain, especially whether optineurin mutations and/or polymorphisms found in patients act as primary or contributing factors in disease pathogenesis. We hope that further insight into how the mechanisms by which optineurin loss- and/or gain-of-function causes different disorders will incite new immunomodulatory therapies.

## Author Contributions

All authors designed, drafted, and approved the final version of the manuscript.

### Conflict of Interest

The authors declare that the research was conducted in the absence of any commercial or financial relationships that could be construed as a potential conflict of interest.
